# Supplementary material for: Analysis of functional brain connectivity in patient with end-stage kidney disease undergoing peritoneal dialysis using functional near infrared spectroscopy
Source: PLoS One. 2025 May 23;20(5):e0323319. doi: 10.1371/journal.pone.0323319 (PMC12101648; doi:10.1371/journal.pone.0323319)
Supplement: S1 Table — (DOCX) [file pone.0323319.s001.docx]

**S1 Table. Correlation analysis of clinical factors and functional brain connectivity**

| Variables | Average degree | Average strength | Eccentricity | Characteristic path length | Global efficiency | Local efficiency | Clustering | Modularity | Assortativity | Small-worldness |
| --- | --- | --- | --- | --- | --- | --- | --- | --- | --- | --- |
| Age | r = –0.115  p = 0.631 | r = 0.096  p = 0.732 | r = –0.490  p = 0.075 | r = –0.108  p = 0.649 | r = –0.096  p = 0.689 | r = –0.025  p = 0.930 | r = 0.096  p = 0.732 | r = –0.139  p = 0.621 | r = 0.011  p = 0.970 | r = 0.477  p = 0.085 |
| MoCA-K | r = –0.036  p = 0.883 | r = 0.115  p = 0.697 | r = –0.377  p = 0.204 | r = 0.101  p = 0.680 | r = –0.075  p = 0.761 | r = 0.185  p = 0.527 | r = 0.156  p = 0.593 | r = –0.121  p = 0.680 | r = 0.198  p = 0.497 | r = 0.551  p = 0.051 |
| Systolic blood pressure | r = 0.097  p = 0.685 | r = –0.016  p = 0.955 | r = –0.119  p = 0.686 | r = –0.078  p = 0.743 | r = 0.108  p = 0.651 | r = –0.129  p = 0.648 | r = –0.161  p = 0.567 | r = 0.029  p = 0.919 | r = –0.282  p = 0.308 | r = 0.062  p = 0.834 |
| Diastolic blood pressure | r = 0.009  p = 0.970 | r = –0.009  p = 0.975 | r = –0.051  p = 0.864 | r = 0.042  p = 0.860 | r = –0.066  p = 0.781 | r = 0.023  p = 0.934 | r = –0.029  p = 0.919 | r = 0.029  p = 0.919 | r = –0.270  p = 0.330 | r = 0.011  p = 0.970 |
| Years of education | r = 0.065  p = 0.790 | r = –0.251  p = 0.386 | r = –0.094  p = 0.760 | r = 0.078  p = 0.751 | r = 0.082  p = 0.739 | r = –0.320  p = 0.265 | r = –0.242  p = 0.405 | r = 0.251  p = 0.386 | r = 0.149  p = 0.611 | r = 0.273  p = 0.367 |
| Dialysis duration | r = –0.234  p = 0.320 | r = –0.301  p = 0.276 | r = 0.316  p = 0.272 | r = –0.038  p = 0.875 | r = –0.259  p = 0.270 | r = –0.351  p = 0.199 | r = –0.136  p = 0.628 | r = 0.183  p = 0.514 | r = 0.143  p = 0.610 | r = –0.148  p = 0.614 |
| Kt/V | r = 0.062  p = 0.796 | r = 0.236  p = 0.396 | r = –0.591  *p = 0.026 | r = –0.165  p = 0.488 | r = 0.125  p = 0.600 | r = 0.206  p = 0.462 | r = 0.156  p = 0.579 | r = –0.152  p = 0.588 | r = –0.199  p = 0.478 | r = 0.498  p = 0.070 |
| Body mass index | r = –0.177  p = 0.456 | r = –0.007  p = 0.980 | r = 0.125  p = 0.670 | r = 0.296  p = 0.205 | r = –0.134  p = 0.574 | r = 0.079  p = 0.781 | r = –0.143  p = 0.612 | r = 0.125  p = 0.657 | r = –0.064  p = 0.820 | r = –0.064  p = 0.829 |
| Albumin | r = 0.046  p = 0.847 | r = –0.054  p = 0.849 | r = 0.060  p = 0.840 | r = –0.009  p = 0.970 | r = 0.138  p = 0.561 | r = 0.111  p = 0.693 | r = –0.068  p = 0.809 | r = 0.179  p = 0.523 | r = –0.057  p = 0.839 | r = –0.148  p = 0.614 |
| Total cholesterol | r = 0.019  p = 0.936 | r = –0.018  p = 0.950 | r = 0.064  p = 0.828 | r = 0.133  p = 0.576 | r = 0.050  p = 0.833 | r = 0.014  p = 0.960 | r = 0.129  p = 0.648 | r = 0.191  p = 0.495 | r = –0.104  p = 0.713 | r = –0.053  p = 0.858 |
| Triglyceride | r = –0.239  p = 0.311 | r = –0.236  p = 0.398 | r = 0.358  p = 0.208 | r = 0.441  p = 0.052 | r = –0.165  p = 0.486 | r = –0.018  p = 0.950 | r = 0.171  p = 0.541 | r = 0.232  p = 0.405 | r = 0.293  p = 0.289 | r = –0.314  p = 0.274 |
| LDL-C | r = –0.127  p = 0.592 | r = –0.265  p = 0.339 | r = 0.333  p = 0.244 | r = 0.221  p = 0.348 | r = –0.111  p = 0.640 | r = –0.276  p = 0.319 | r = –0.022  p = 0.939 | r = 0.323  p = 0.241 | r = 0.029  p = 0.919 | r = –0.355  p = 0.212 |
| HDL-C | r = 0.298  p = 0.202 | r = 0.246  p = 0.376 | r = –0.218  p = 0.455 | r = –0.278  p = 0.235 | r = 0.267  p = 0.255 | r = 0.139  p = 0.621 | r = –0.132  p = 0.639 | r = 0.004  p = 0.990 | r = –0.589  *p = 0.021 | r = 0.266  p = 0.358 |
| Hemoglobin | r = 0.060  p = 0.802 | r = 0.493  p = 0.062 | r = –0.460  p = 0.098 | r = –0.247  p = 0.293 | r = 0.111  p = 0.640 | r = 0.502  p = 0.056 | r = 0.474  p = 0.075 | r = –0.517  *p = 0.049 | r = 0.245  p = 0.379 | r = 0.396  p = 0.161 |
| Iron | r = 0.142  p = 0.552 | r = 0.275  p = 0.321 | r = –0.174  p = 0.553 | r = –0.299  p = 0.200 | r = 0.062  p = 0.796 | r = 0.318  p = 0.248 | r = 0.443  p = 0.098 | r = –0.368  p = 0.177 | r = 0.339  p = 0.216 | r = 0.103  p = 0.725 |
| Ferritin | r = –0.036  p = 0.880 | r = –0.089  p = 0.752 | r = 0.116  p = 0.692 | r = –0.134  p = 0.574 | r = –0.135  p = 0.569 | r = –0.132  p = 0.639 | r = 0.043  p = 0.879 | r = 0.054  p = 0.850 | r = –0.139  p = 0.621 | r = –0.007  p = 0.982 |
| TIBC | r = 0.118  p = 0.622 | r = 0.064  p = 0.820 | r = –0.090  p = 0.759 | r = 0.033  p = 0.890 | r = 0.278  p = 0.236 | r = 0.199  p = 0.478 | r = 0.023  p = 0.934 | r = –0.097  p = 0.732 | r = 0.100  p = 0.722 | r = 0.068  p = 0.817 |
| Transferrin saturation | r = 0.085  p = 0.721 | r = 0.121  p = 0.666 | r = –0.112  p = 0.703 | r = –0.268  p = 0.254 | r = –0.008  p = 0.975 | r = 0.146  p = 0.603 | r = 0.371  p = 0.173 | r = –0.200  p = 0.475 | r = 0.275  p = 0.321 | r = 0.055  p = 0.852 |
| Parathyroid hormone | r = –0.154  p = 0.516 | r = –0.332  p = 0.226 | r = 0.530  p = 0.051 | r = 0.284  p = 0.225 | r = –0.202  p = 0.394 | r = –0.200  p = 0.475 | r = –0.332  p = 0.226 | r = 0.364  p = 0.182 | r = –0.218  p = 0.435 | r = –0.358  p = 0.208 |
| Calcium | r = 0.261  p = 0.267 | r = 0.290  p = 0.295 | r = –0.447  p = 0.109 | r = –0.430  p = 0.058 | r = 0.317  p = 0.174 | r = 0.302  p = 0.273 | r = 0.308  p = 0.265 | r = –0.147  p = 0.602 | r = –0.222  p = 0.427 | r = 0.352  p = 0.217 |
| Phosphate | r = –0.045  p = 0.851 | r = –0.481  p = 0.070 | r = 0.669  *p = 0.009 | r = 0.170  p = 0.473 | r = –0.074  p = 0.757 | r = –0.465  p = 0.081 | r = –0.399  p = 0.141 | r = 0.481  p = 0.070 | r = –0.029  p = 0.919 | r = –0.535  p = 0.049 |

LDL-C: Low-density lipoprotein cholesterol, HDL-C: High-density lipoprotein cholesterol, TIBC: Total iron-binding capacity

*Statistical significance (*p* < 0.05)

Several clinical factors were significantly correlated with functional brain connectivity measures, including Kt/V and eccentricity (r = –0.591, p = 0.026), high density lipoprotein cholesterol (HDL-C), assortativity (r = –0.589, p = 0.021), hemoglobin and modularity (r = –0.517, p = 0.049), phosphate and eccentricity (r = 0.669, p = 0.009), and phosphate and small-worldness (r = –0.535, p = 0.049)
