# Supplementary material for: Analysis of functional brain connectivity in patient with end-stage kidney disease undergoing peritoneal dialysis using functional near infrared spectroscopy
Source: PLoS One. 2025 May 23;20(5):e0323319. doi: 10.1371/journal.pone.0323319 (PMC12101648; doi:10.1371/journal.pone.0323319)
Supplement: Attachment — Submitted filename: response to review.docx [file pone.0323319.s003.docx]

We would like to thank for the editor and reviewers of the *PLOS One* for taking their time to review our article. We made some corrections and clarifications in the manuscript after going over the reviewers’ comments, again.

We hope the revised manuscript will better meet the requirements of the *PLOS One* for publication. We would like to thank you once again for the constructive review by the reviewers and editor.

 #**Reviewer 1**

The article is a study examining the neurological effects and brain connectivity in peritoneal dialysis patients, and it is one of the conditions that is frequently encountered in daily practice and is not always easy to manage. I have a few suggestions for this interesting study.

The patients were evaluated by a neurologist, Were any imaging modalities done? (CT? MRI?) I did not see any additional information in this regard. Especially since prefrontal area data can be collected, have there been tests that can show dysfunction of this domain? Were vascular examinations performed in both groups?

: Thank you for your wonderful review. Unfortunately, our study did not include imaging modalities such as CT or MRI. However, patients with any prior neurological or psychiatric diseases were excluded from the experimental group. If imaging tests had been conducted, we believe we could have more definitively ruled out neurological issues. This is discussed in the limitations.

The MoCA-K used in this study is a test that reflects dysfunction in the prefrontal area. While it is a screening tool for cognitive impairment, it includes several items that assess executive function and attention, which can reflect the function of the frontal lobe. We recruited patients without a neurological history, and the average MoCA-K score was 24, which is above the threshold of 22 that suggests cognitive impairment, and no correlation with functional brain connectivity was observed. Vascular examination was not conducted in either group, which is also mentioned as a limitation.

I was able to examine the biochemistry values of the patient group, but I could not see the healthy group. Was it not looked at or shared? It is probably planned to increase the number, but I think it would strengthen the hypothesis if there was a third group receiving HD. I believe that these feedbacks should be made.

: The control group consisted of individuals without underlying diseases and with no health issues. Since the study aimed to investigate the correlation between laboratory data and functional brain connectivity in patients with ESKD undergoing PD, laboratory data from healthy controls were not collected.

By measuring changes in functional brain connectivity using fNIRS in ESKD patients undergoing hemodialysis and comparing them, it would be possible to better understand the mechanisms through which PD affects neurological complications in ESKD patients in comparison to HD. I agree with your opinion, and this has been added as a limitation in the study.

**#Reviewer 2**

This is an interesting manuscript that gives us more information about the potential mechanisms of cognitive decay among people with end-stage kidney disease (ESKD) undergoing PD. The study is well conceived, but in my view, comparing people undergoing PD with healthy controls may overestimate the loss of brain connectivity. This could be addressed by evaluating people with ESKD at the onset of PD and then at pre-established time points, hence making each participant her or his control. As it is unlikely to be feasible to recruit and follow up participants for extended periods, it should be mentioned as a limitation and a window for follow-up studies.

: Thank you for your excellent review. I have added the additional points to the limitations section based on your advice.
